# Palestinian university students’ perspectives on COVID-19 risk and remote learning during the pandemic: A qualitative photovoice study

**DOI:** 10.1371/journal.pone.0311972

**Published:** 2024-10-11

**Authors:** Mohammed B. A. Sarhan, Hanin Basha, Rita Giacaman, Masamine Jimba, Rika Fujiya

**Affiliations:** 1 Graduate School of Health Management, Keio University, Fujisawa, Kanagawa, Japan; 2 Department of Community and Global Health, Graduate School of Medicine, The University of Tokyo, Bunkyo-ku, Tokyo, Japan; 3 Institute of Community and Public Health, Birzeit University, Birzeit, Palestine; 4 Faculty of Nursing and Medical Care, Keio University, Fujisawa, Kanagawa, Japan; Qatar University, College of Health Sciences, QATAR

## Abstract

**Background:**

The COVID-19 pandemic has triggered major changes worldwide, with repercussions on mental health and education. The present study primarily aimed to retrospectively explore undergraduate students’ risk perceptions of COVID-19 and their experiences with remote learning during the pandemic, with an emphasis on their high school years.

**Methods:**

This qualitative photovoice study was conducted between 30 March and 4 May 2023. The target population of this study was first- and second-year undergraduate students who had been in high school during the pandemic. Maximum variation, snowball, and convenience sampling methods were used to recruit students. They were asked to share photos and provide comments in written or voice messages explaining their experiences during the pandemic. Voice messages were manually transcribed verbatim. A manual content analysis of these comments was performed to derive the codes and themes relevant to the study objectives.

**Results:**

Forty-seven students initially agreed to participate and signed an informed consent form. However, the final sample included 16 students (nine men and seven women). The content analysis revealed four themes that were identified as the main factors contributing to high school students’ risk perceptions of COVID-19. The first theme was psychosocial factors related to the pandemic, including emotions, isolation and stress. The second focused on the mechanisms used to cope with the pandemic. The third theme included beliefs and behaviours that either increased or decreased COVID-19 risk. The final theme addressed schools’ responses to COVID-19, including factors such as maintaining connections with schools, preventive measures and the transition to remote learning.

**Conclusion:**

This study highlighted the extensive impact of the pandemic on Palestinian high school students, demanding instantaneous adaptation to ensure their safety and well-being while maintaining the quality of education. Remote learning has become an important strategy with opportunities and challenges for high school students.

## Introduction

As of 14 July 2024, over than 775 million cases of coronavirus disease 2019 (COVID-19) had been identified with more than 7 million deaths [1]. The COVID-19 pandemic had triggered significant global changes [2, 3] that significantly and negatively affected people’s daily life lifestyle, routine and physical and psychological well-being [3–5]. Furthermore, the extended lockdowns affected people’s transportation and freedom of movement, thereby leading to economic hardships [3]. The pandemic caused multiple adverse effects in various sectors and socioeconomic activities in addition to its major impact on the health sector [3, 6]. Globally, the COVID-19 pandemic disrupted major sectors such as global trade and supply chains, agriculture and food, automotive, aviation and transportation, travel and tourism, sports and entertainment [6]. Therefore, understanding the population’s response to the COVID-19 crisis is crucial [3].

The COVID-19 pandemic has particularly affected the mental health and academic progress of high-school students. This is attributed to the preventive measures, especially lockdowns, imposed to fight the rapid transmission of COVID-19 [2]. It has contributed to massive mental health issues, especially among young people [7–9]. These mental health issues have been correlated with adolescents’ and young adults’ concerns about being infected with COVID-19 and changes in social life (e.g. staying at home) [7]. College students reported increased feelings of loneliness during the pandemic, especially at the beginning. This feeling is a result of reduced or disrupted social support due to the pandemic [8]. Lee et al. reported that loneliness was a major factor in the appearance of depressive symptoms among young adults [8]. However, in other cases, lockdown was associated with lower social anxiety symptoms, indicating that the pandemic provided an escape from the burden of social pressures. Additionally, school-related challenges were strongly associated with mental health issues during the COVID-19 pandemic. Depressive symptoms were reported to be higher among students who had negative perceptions of school, such as passing exams, finishing assignments, and the quality of online classes [7].

Schools are responsible for providing nurturance, learning, and the knowledge and skills required to become a well-educated and healthy individual [10]. They are also responsible for promoting health and preventive behaviours through health education activities [11]. During COVID-19, a transition from traditional in-person learning to remote learning was required to maintain these fundamental school functions [2]. The main advantage of remote learning is that it sustains the process of education and learning [12]. However, the sudden transition towards remote learning without adequate preparation of students or teachers has reduced its acceptance among students [13, 14], along with other challenges such as educational style and technical issues in educational institutions [14, 15]. Online classes have been a major stressor for young people during the pandemic [7].

Providing the correct knowledge about pandemics is essential for shaping people’s perceptions of accepting and following preventive behaviours [16, 17]. Perceptions are subjective assessments of critical events that influence people’s actions to reduce the risk of exposure. This applies to COVID-19 and other pandemics such as the H1N1 flu [18]. Perceptions of the COVID-19 pandemic were influenced by social and cultural factors, personal experiences [19] and the credibility of information sources [20]. Acquired knowledge and perceptions can be communicated using different approaches, including social media, which are easily accessible to the public [21].

Several studies have assessed high school students’ perceptions of the risk of COVID-19 in the Middle East. Egyptian medical and Libyan college students reportedly had a high level of understanding and perception of the COVID-19 risk [22, 23]. Other studies in Jordan, Saudi Arabia and Turkey reported low to moderate risk perception levels [24–26]. Globally, a study in the Netherlands reported that young adults have low personal and high impersonal (i.e. towards others) risk perception [27]. In China, college students reported a high-risk perception of COVID-19, which might be necessary for delivering health information and warnings to them [28]. Furthermore, in Spain and Ireland, students with a high-risk perception of COVID-19 were more likely to follow the recommended preventive measures [29, 30]. In Palestine, one study assessed dental students’ risk perception towards COVID-19. In this study, most dental students reported moderate risk perceptions of providing dental care at university clinics [31].

In Palestine, an initial lockdown was announced for thirty days in early March 2020 [32]. Thereafter, school closures were announced leaving 1.3 million students, including more than 260,000 high school students, without a school [33, 34]. However, by 10 March 2020, the Palestinian Ministry of Education decided to transition to remote learning [33]. The educational system in Palestine may have amplified mental health problems caused by the COVID-19 pandemic. Among the sufferers, high school and undergraduate students in Palestine showed higher chances of being distressed than other groups [35, 36]. In Palestine, the educational system includes a critical transition phase marked by the “Tawjihi” or the national high school examination, which twelfth-grade high school students must pass as a determinant for their future educational and career choices [37]. While several studies on COVID-19 have utilised the photovoice approach, such as those in Malawi, Bolivia and Indonesia [38–40], only one has addressed COVID-19 risk perception [38]. In Malawi, religious and political beliefs have been found to be the main factors undermining the risk perception of COVID-19 [38]. Moreover, previous research in Palestine and the Arab World did not specifically employ this approach to assess students’ risk perceptions of the COVID-19 pandemic. Therefore, the present study primarily aimed to retrospectively explore undergraduate students’ risk perceptions of COVID-19 and their experiences of remote learning during the pandemic, with emphasis on their high school years. The uniqueness of this study lies in its ability to contribute to the understanding of the long-term impact of COVID-19.

## Methods

### Study design and setting

The present study aimed to explore students’ lived experiences and perspectives on the risk of COVID-19 and transition towards remote learning during the pandemic. Therefore, qualitative design using a photovoice approach was adopted. Data was collected between 30 March 2023 and 4 May 2023 at a Palestinian University located in Ramallah and Al Bireh District. The University had over 14,000 students enrolled in 131 undergraduate and graduate programs.

### Theoretical framework

The present study was based on the social ecological model of health [41, 42] that considers health to be the result of the interaction between individuals and their community and physical, social and political environments [41]. The first level refers to individual or intrapersonal demographic and socioeconomic characteristics along with the individual’s beliefs, knowledge, skills, attitudes and behaviours. The second level incorporates formal and informal interpersonal social relations and interactions with family, friends and partners, which can influence people’s experiences and behaviours. The third level highlights understanding the role of institutions and organisations in spreading health-promoting messages and supporting behavioural changes. It further emphasises the social connections and interactions that occur with surrounding communities and settings to which people belong, such as schools, neighbourhoods or workplaces. The final level comprises broader societal factors, represented by health, economic, educational and social policies that either impair or improve the population’s health [41, 42].

### Photovoice

Photovoice is a qualitative research method in which participants are asked to express their perceptions, opinions and thoughts on a certain issue through taking photographs and providing an explanation on them. This approach can empower youths to become part of the solution to public health and social problems in their communities [43].

Following this approach, the students were asked to share two photographs: one photograph to describe their perception of COVID-19 and one to describe their perception of remote learning. Participants were required to provide a description of what was shown or what was happening in the photographs. Considering that some photos may not be self-explanatory and may be difficult for the researchers to understand without descriptions, some questions were provided to the students to assist them in elaborating on the photos and explain how the photos expressed their experiences during COVID-19. Additionally, they were required to answer the following questions based on their experiences and photographs:

1. Perceptions towards COVID-19 pandemic:
  - How does this photo relate to your COVID-19 risk perception and preventive behaviours?
  - How does this photograph describe the role of your educational institution in preventing the spread of COVID-19?
2. Perceptions towards remote learning during the pandemic:
  - How does this photograph illustrate the impact of remote learning on academic achievement and performance?
  - How does this photograph explain the effectiveness and/or barriers to and challenges of remote learning during the lockdown, especially in high school?
  - How does this photograph illustrate the impact of remote learning on one’s mental health, social life and lifestyle?
  - What do you think should be done to overcome the barriers and challenges of remote learning? Provide some suggestions for improvement.

Students were required to provide their responses via WhatsApp or email, leveraging the widespread use of social media and text-messaging platforms among the youth. This choice was grounded in the confidentiality, accessibility, ease, convenience and low cost of these communication channels. Additionally, using WhatsApp or email afforded students the opportunity and time to carefully consider their responses before submission, thereby enhancing the quality and depth of their responses [44].

Initially, the students had to submit a written response, but the research team later decided to give them the freedom to share their responses as voice messages via WhatsApp at their convenience. This decision was made to accommodate students who preferred verbal communication over writing. The research team sought to reduce potential dropouts or withdrawals by giving the students the choice of how to deliver their responses, thereby addressing this concern raised by some students during the recruitment process.

Students were asked to select photos that could have been taken at any time, as long as they were able to link them to the research themes and objectives. These photos could have been taken for research purposes or by students before (e.g. during the lockdown). Furthermore, the students were given one week to submit their photos. In case a participant missed the deadline, a reminder was sent to them by HB or MBAS.

### Recruitment

This study targeted first- and second-year university students (primarily high-school students during the COVID-19 pandemic). Students were excluded if they refused to provide their consent, failed to submit the photos on time or provided photos that were not compatible with the study’s objectives. Of the 47 students who initially agreed to participate and provided their consent, 16 completed the required task.

Researchers (MBAS, PhD, male; HB, female, Masters; RF, female, PhD) with experience in conducting qualitative studies decided to follow various approaches to recruit students: (1) visiting various faculties during break time and approaching students, (2) asking for the help of academic and administrative employees at the faculty’s office, libraries, and student affairs departments, (3) contacting lecturers and student counsellors, (4) conducting snowball sampling by asking students to guide us to their friends, and (5) posting an announcement on the students’ academic portal. Based on an agreement with the research team represented by the second author (HB), the university student affairs department agreed to provide students who joined the study with an incentive of ten hours in their voluntary work records. Completion of a specific number of voluntary working hours is a requirement for graduation. These hours could be spent providing voluntary services at the community level, such as health, environment, agriculture (e.g., olive picking), education and culture. Eventually, maximum variation (in terms of gender, discipline and place of residence), snowball sampling and convenience sampling methods were used to recruit students.

### Data analysis

The data analysis was initiated even as the recruitment process continued. The researchers stopped recruiting additional students because the data reached the point of sufficiency and saturation and did not reveal new insights. According to previous studies, saturation can be reached in fewer than 25 interviews (9 and 17 interviews, on average) [45]. HB transcribed the voice notes into Arabic, and MBAS, and HB separately translated the written and transcribed explanations and responses into English. MBAS and HB followed the content analysis method separately to manually extract codes and themes [46]. Both MBAS and HB extracted meaning units from students’ responses, condensed them into smaller phrases while maintaining their original meanings and extracted common codes from them. Finally, MBAS and HB selected the main themes, which were further refined and conciliated by them to obtain the final themes. Subsequent revisions were made based on the feedback from the fourth (MJ) and last (RF) authors. The data were reported based on the consolidated criteria for reporting a qualitative research (COREQ) checklist.

### Ethical consideration

Ethical approval was obtained from the Research Ethics Committee of the Institute of Community and Public Health at Birzeit University in Palestine (reference number: 2023 [1–3]). Permission to conduct this research was obtained from the Faculty of Nursing and Medical Care, Keio University, in March 2023 (permission number: Outer 2022–10). The research team disposed student’s personal information, including e-mail and phone numbers needed for WhatsApp, after the completion of data analysis.

Students voluntarily completed a written consent form if they were interested in participating. Following completion, the HB signed the form. The consent form provided a description of the photovoice approach and study objectives. HB clearly explained the ethical considerations regarding photos to students through verbal and written instructions (distributed to the participants). Additionally, students received instructions on the ethical considerations regarding the photos they chose to share. For example, students must seek verbal permission from anyone whose face appears in a photo. Students can keep photos; however, they must not upload them to any social media platform. Furthermore, they understood that the research team had the right to use the photos for any future publication. During data analysis and manuscript writing, MBAS blurred the faces in the photos to prevent their identification.

## Results

### Characteristics of the participants

Among the 16 students recruited in this study, seven were women and nine were men. The recruited students were from Ramallah and East Jerusalem in the centre and Hebron in the south of the West Bank. Most students were aged between 18 and 20 years. They were first- and second-year university students. The majority belonged to scientific faculties, including computer science, nutrition, computer engineering, physics, accounting, audiology and speech therapy, while one student belonged to a cultural studies major. The participants’ characteristics are shown in S1 Table.

### Psycho-social factors

#### Emotions related to the COVID-19 pandemic

During the pandemic, students experienced a mix of negative feelings such as panic, stress and tension. These feelings were mainly due to the fear of being infected with COVID-19 infection. They were also attributed to the fear of infecting susceptible family members after contact with infected colleagues in high school.

*We could not proceed with our study activities because of the presence of four or five infected students who were unaware of their status. This situation led to repeated quarantines and increased anxiety. We were afraid of the transmission of COVID-19 to vulnerable family members.* (S1)

#### Disruption of social life

Disruption of the social lives of high school students was a major contributor to their mental health status. Being isolated from their social circles was an unpleasant experience that led to the development of feelings of loneliness. Students expressed great concern about losing their ability to maintain friendships and social relationships. Learning about the deaths of their relatives and not being able to support their family were some of the worst experiences.

*All these factors sometimes led to feelings of depression, which became clear after the end of the pandemic and the return to normal life, as I became accustomed to loneliness and isolation and was unable to reestablish friendships or even acquaintances.* (S3)

#### Stress related to the educational process

**Fig 1:**
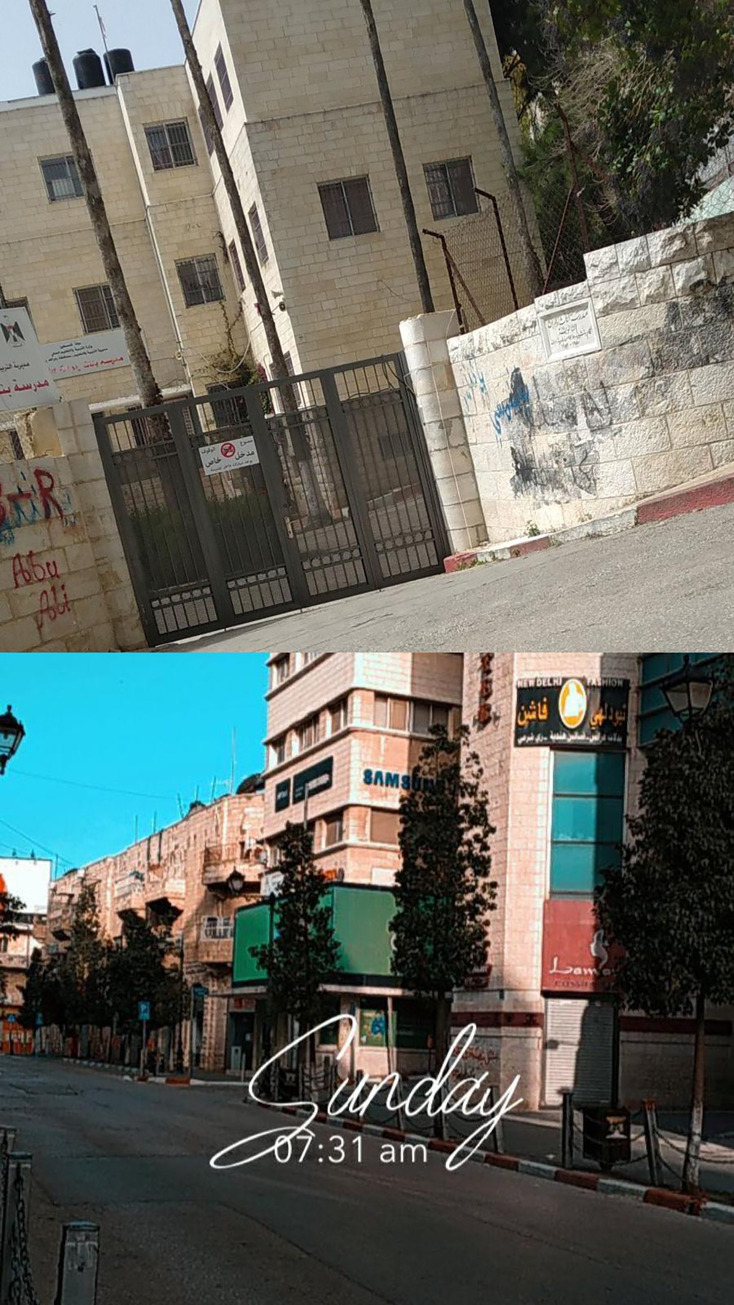
*Upper*: A high school where Tawjihi students had to attend classes during the pandemic. Lower: Empty city during the lockdown while Tawjihi students had to attend classes face to face.

Other factors that served as stressors for students included education and preparation for high school examinations. Many students were in their final high school years (Tawjihi) during the pandemic. Since Tawjihi was extremely important to them in determining their academic future, they were excluded from the lockdown and had to attend school (Fig 1). Therefore, students experienced extreme stress due to the pandemic and Tawjihi at the same time.

*In light of the COVID-19 pandemic, high school has had a significant impact on my psychological status and education.* (S2)

### Tools and mechanisms to cope with COVID-19

**Fig 2:**
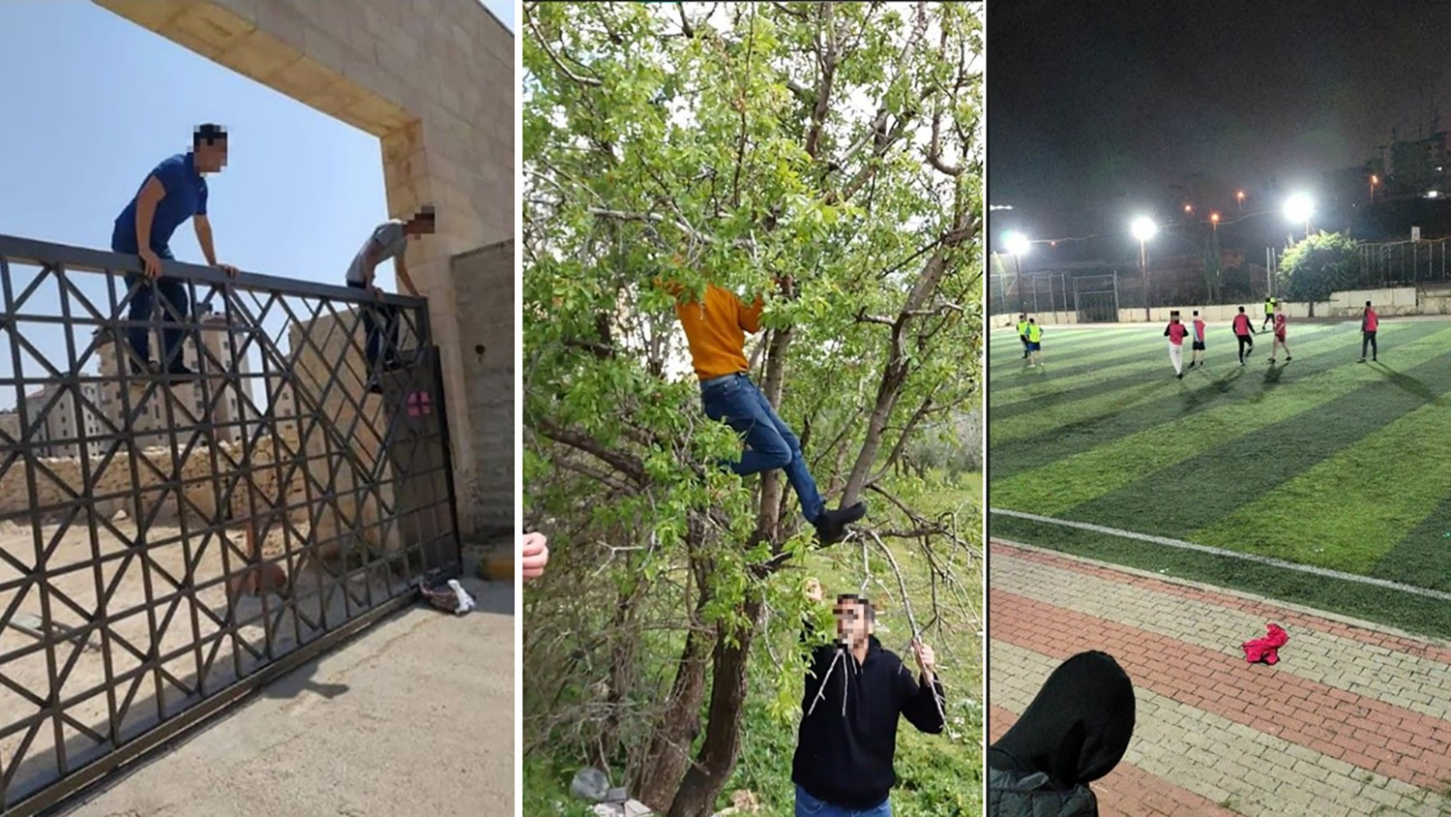
*Left*: Climbing the door of a national park that was closed due to the pandemic. *Middle*: Climbing neighbourhood trees to eat some of its fruits. *Right*: Playing football in one of the playgrounds.

Students reported several ways of coping with the pandemic and the associated stressors and negative emotions. Some students reported spending time with family members, friends or neighbours to recreate regular social experiences (Fig 2).

**Fig 3:**
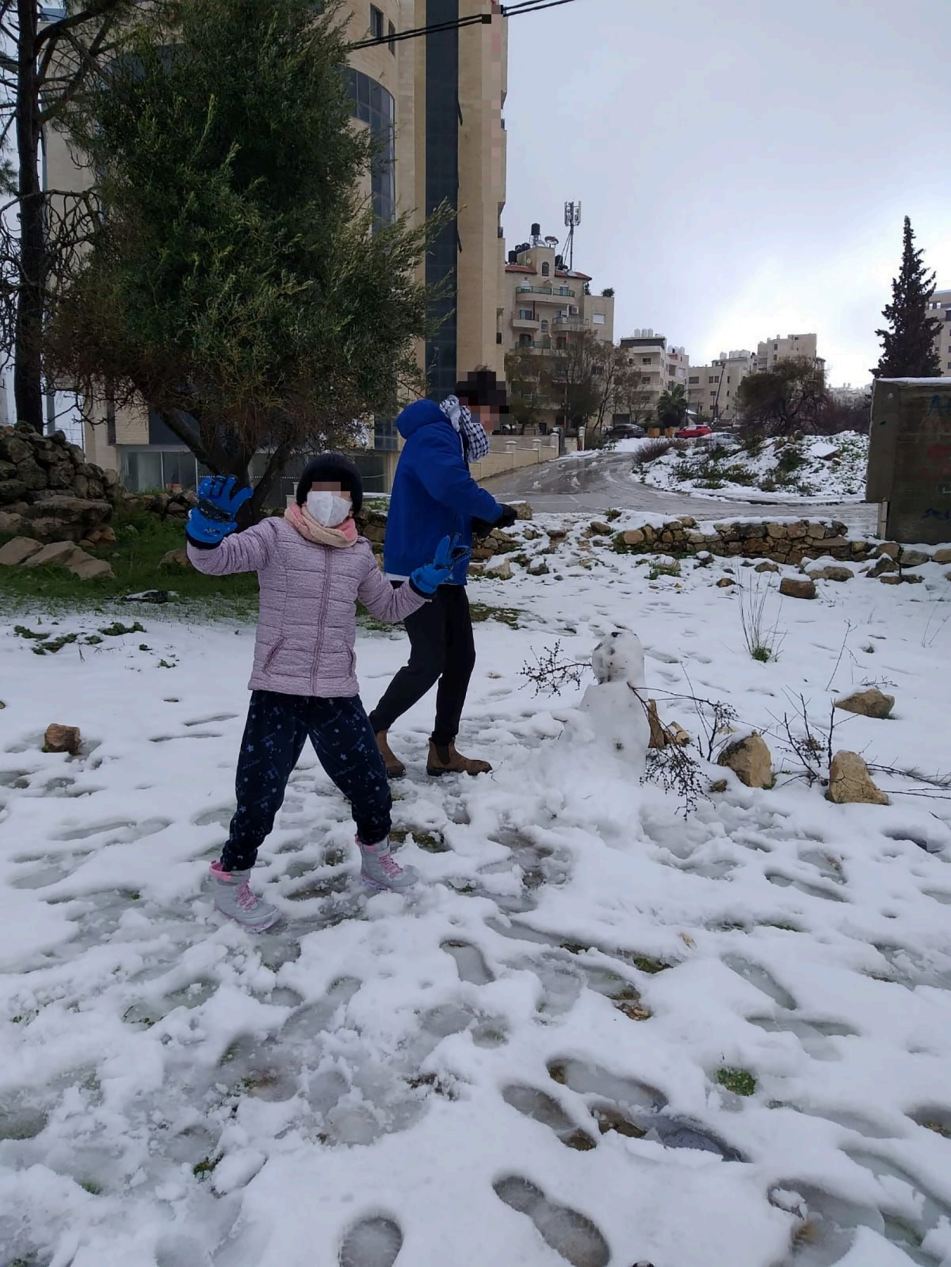
Siblings enjoy playing with snow during the pandemic.

Socialising with others seemed important for students to relieve their stress related to the lockdown or quarantine after a family member was infected. Some of them engaged in activities while following preventive measures, such as wearing masks or social distancing (Fig 3).

**Fig 4:**
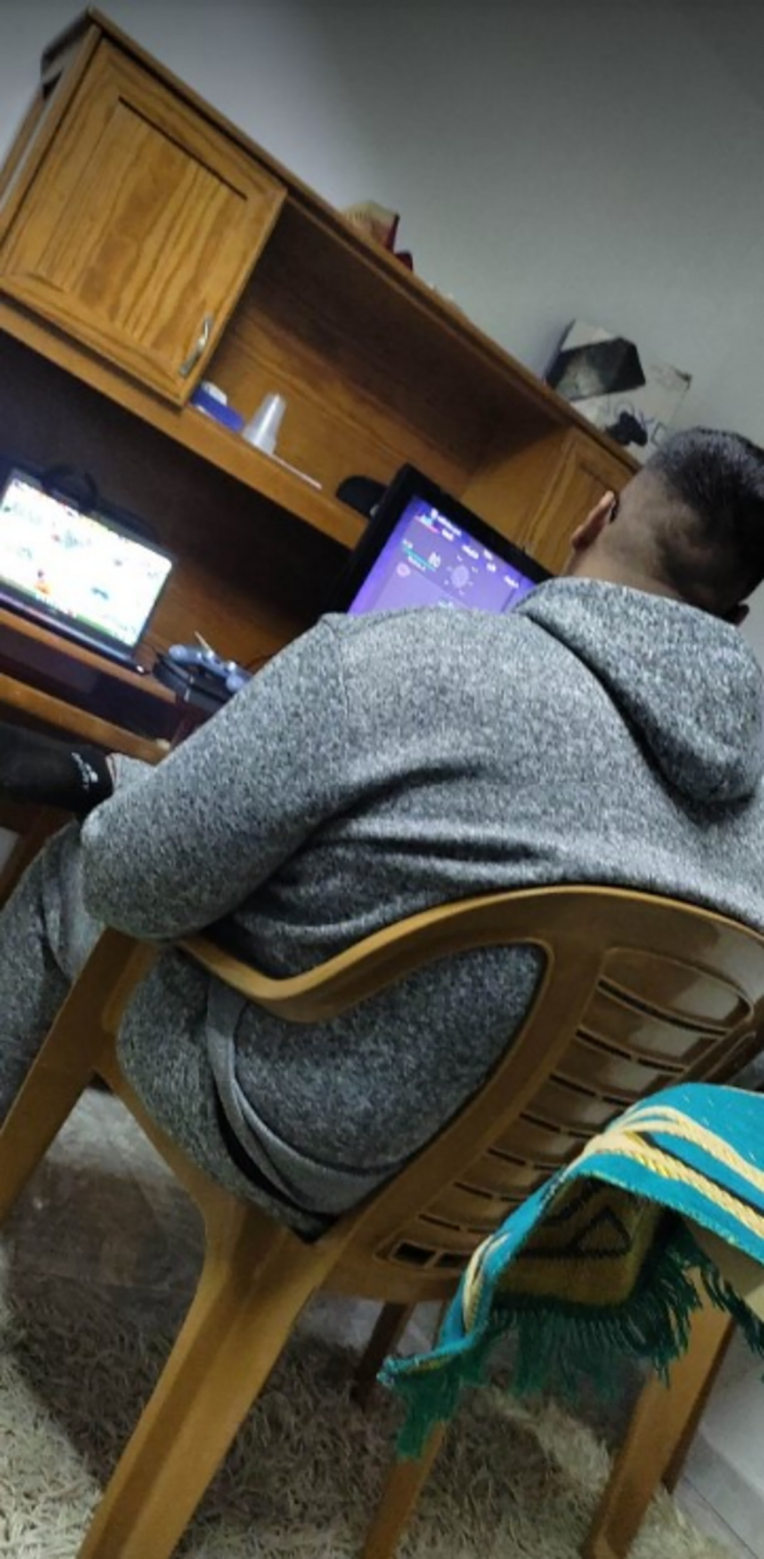
A student playing video games.

**Fig 5:**
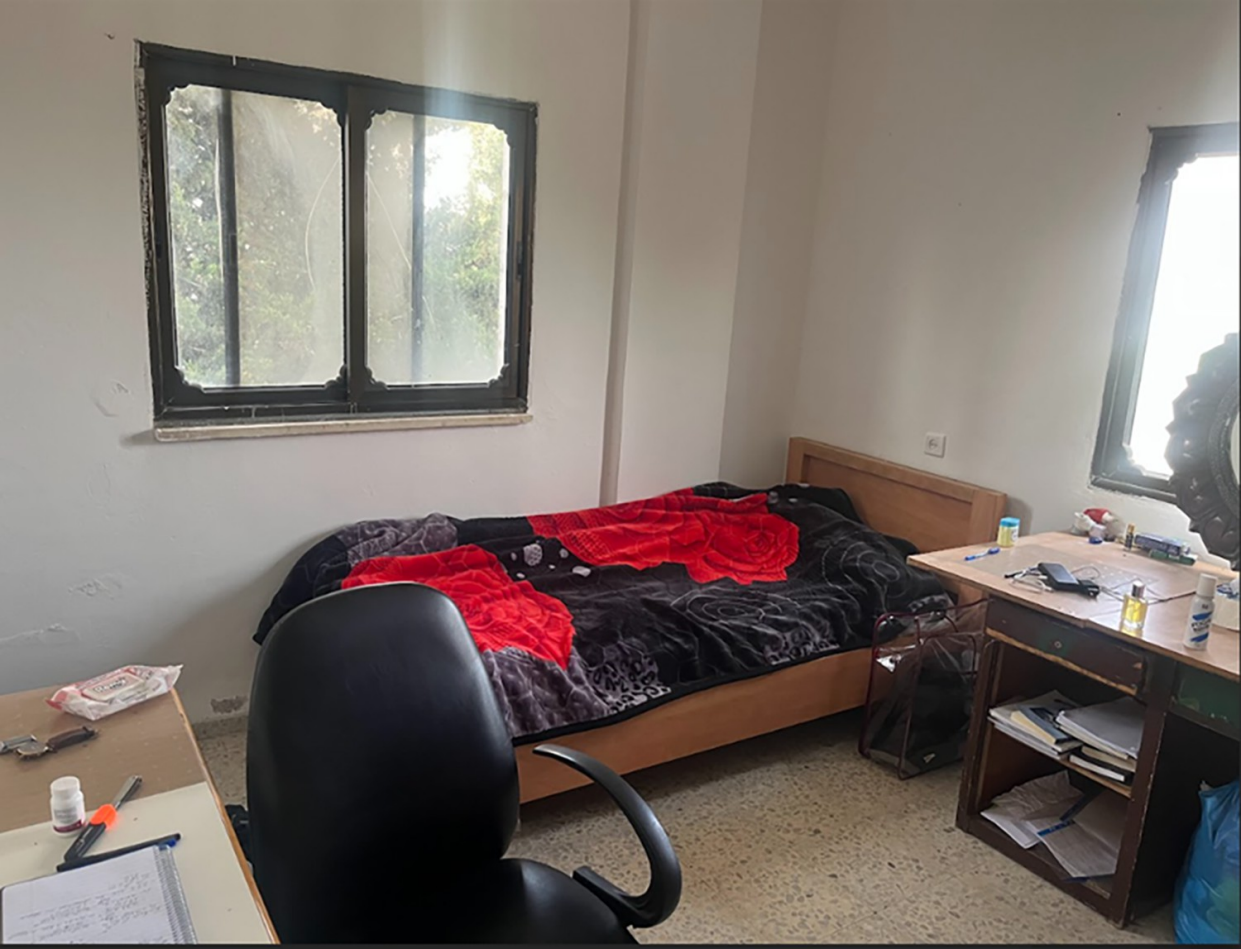
A student’s room where he spent most of his time during the pandemic.

The Internet, entertainment websites, social media and video games were useful for escaping and overcoming the psychological impact of the lockdown. Students found these activities helpful for entertainment and to keep in touch with their social circles during this difficult situation (Fig 4). However, addiction was an immediate consequence of excessive use of these sites and video games. One student reported suffering from loneliness and social isolation due to an addiction to video games (Fig 5).

*I confined myself to prison to protect myself from the pandemic and found nothing to fill in my free time except for the Internet and games. I became addicted to these sites, which required considerable time. This addiction also led to loneliness and isolation, which, in turn, destroyed my social life. I was unable to establish social relationships with my peers.* (S3)

**Fig 6:**
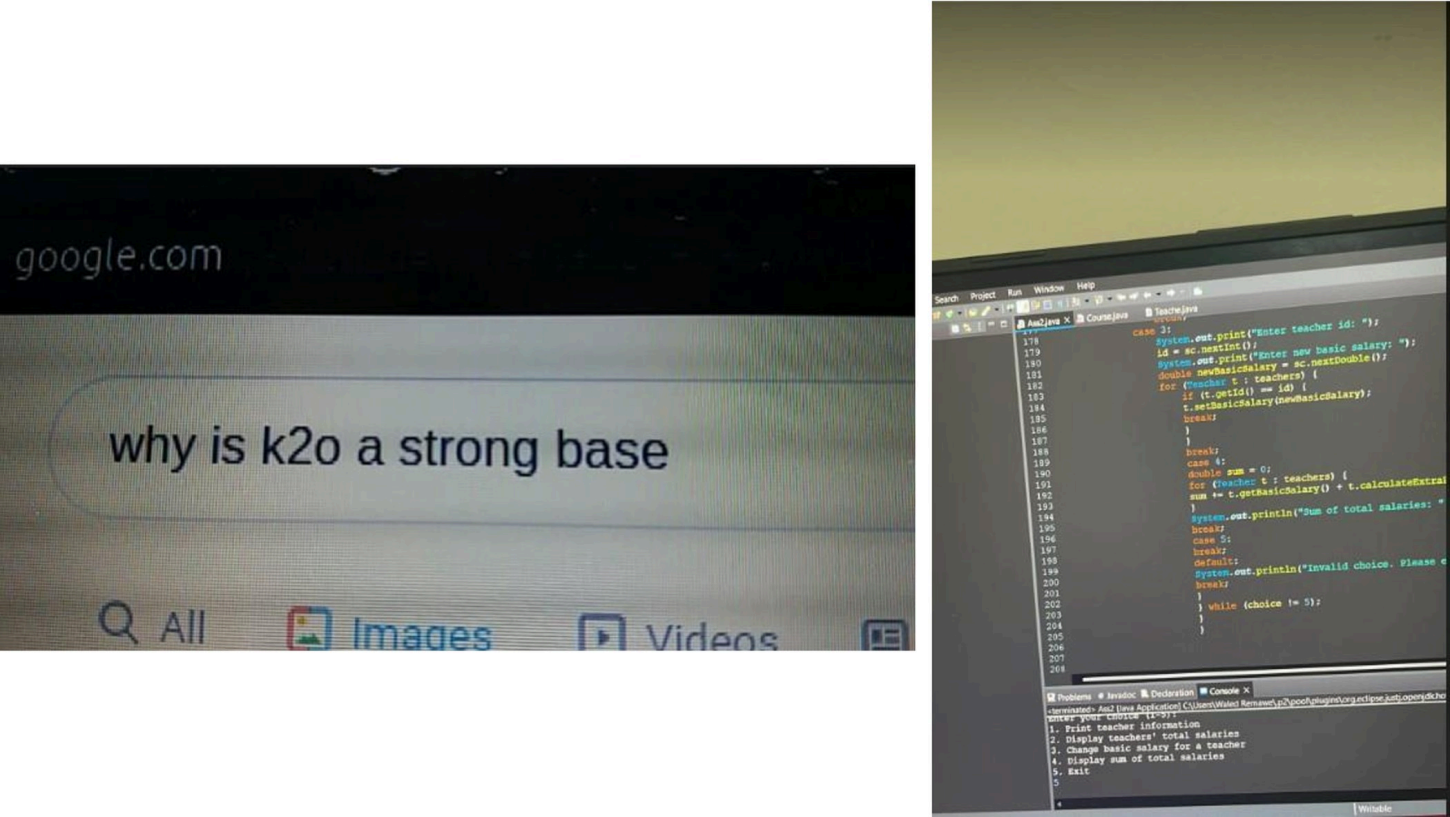
*Left*: A student’s Google search while studying. *Right*: A student learning programming during the pandemic.

On the other hand, several students used lockdown time and the Internet to help them understand and study their school materials, while others used it for self-development, such as developing new computer skills (e.g. programming) (Fig 6).

### Beliefs and behaviours related to COVID-19 risk

#### COVID-19 risk increasing beliefs and behaviours

**Fig 7:**
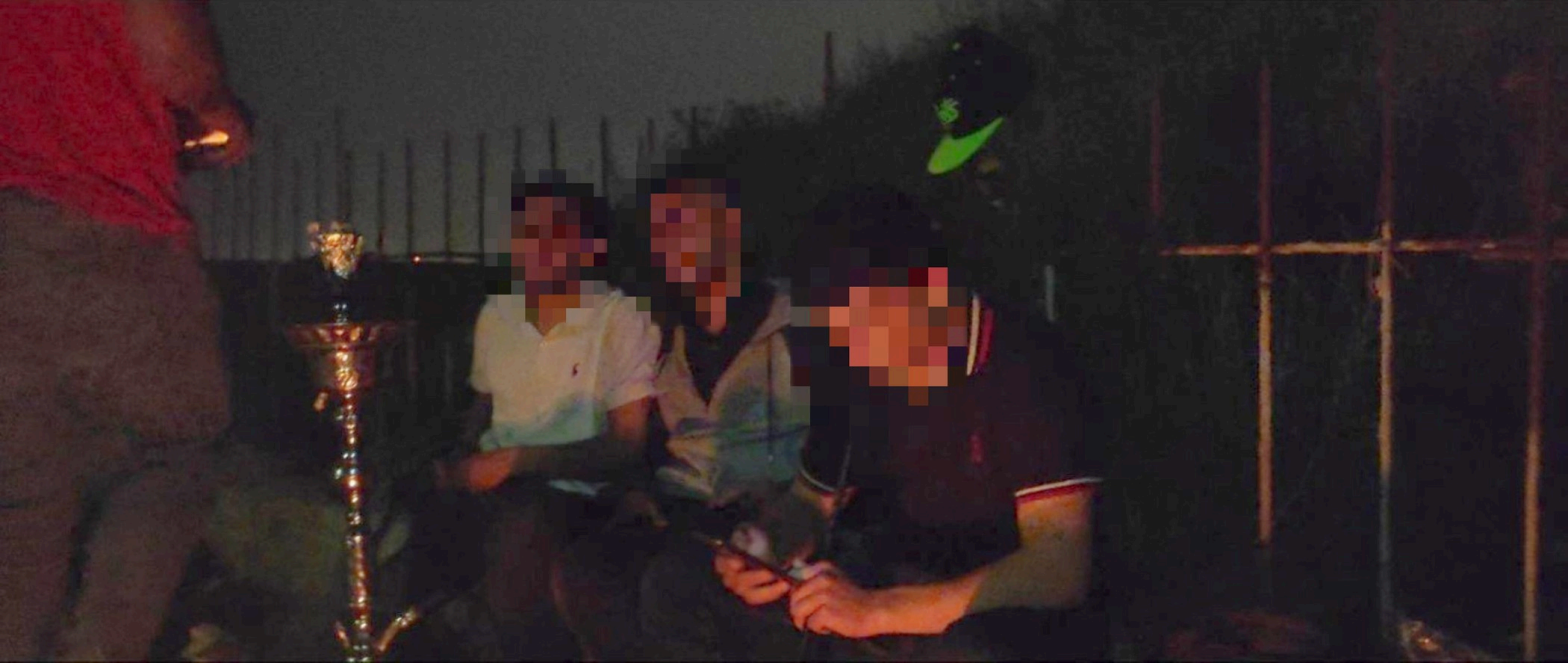
Students gathering and smoking a water pipe during the lockdown.

Students reported various risky attitudes and behaviours, leading to the spread of COVID-19. They were not dedicated to following the lockdown protocols, wearing masks, sharing their belongings, or attending crowded events. The results are shown in Figs 2, 3 and 7. Many students reported low or no concern about the pandemic since they did not consider it to be a serious issue.

An unclear understanding of the pandemic might explain some students’ carelessness in following the preventive measures. Another student reported that he liked staying at home as he considered it as a vacation.

*Of course, we did not have a real understanding of the meaning of a global pandemic that could spread rapidly and have many negative effects on people, but we were happy to be on vacation.* (S4)

#### COVID-19 risk reduction beliefs and behaviours

**Fig 8:**
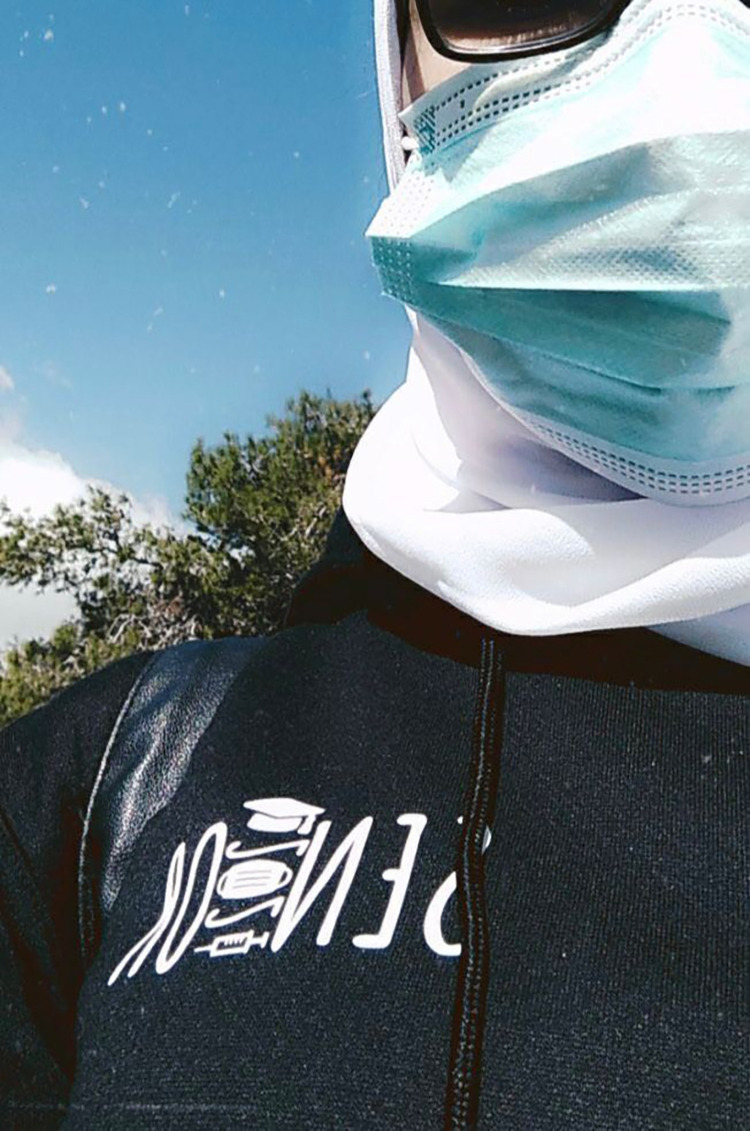
A student wearing mask while walking outside.

Some students reported having knowledge and awareness of behaviours that contributed to the spread of the pandemic. They believed in the importance of committing to preventive behaviours, such as wearing masks, avoiding crowded areas, avoiding using others’ belongings, and staying at home (Fig 8).

*I think the level of awareness among the surrounding community was high as everyone was taking precautions against COVID-19 by wearing masks and constantly using sanitisers.* (S8)

Furthermore, some students showed a sense of responsibility not only towards themselves but also towards others.

*My understanding of COVID-19 was that I needed to stay at home to provide space for the relevant authorities to perform their jobs and protect my family from transmitting the infection to them.* (S7)

**Fig 9:**
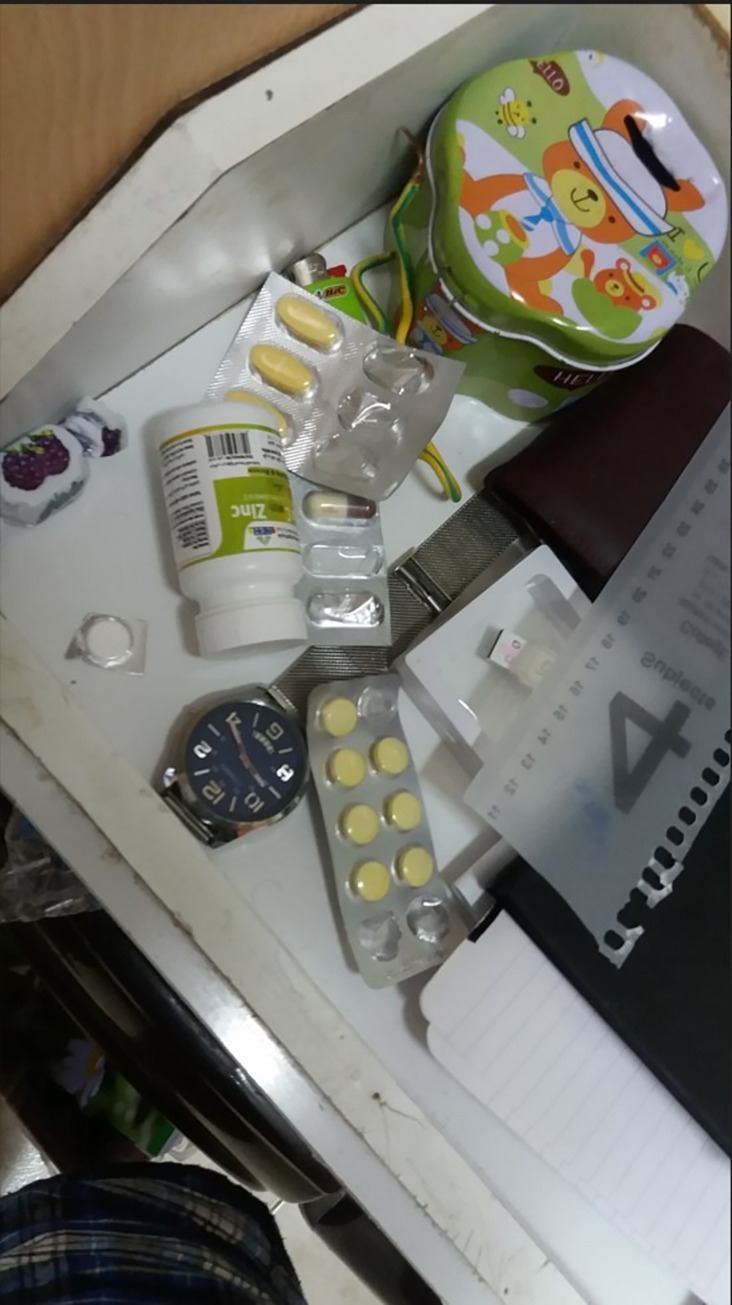
Various medications were taken by the student during the pandemic forced by his parents.

However, another student reported suffering from the extreme precautions imposed on him by his family (Fig 9):

*Their (parents) concern for precautionary measures was excessive to the point of being annoying, with regular sterilizations and taking medications.* (S13)

### Schools’ response to the COVID-19 pandemic

#### Maintaining connections to support students

Students in the present study considered the lack of continuous communication, interaction and guidance from schools and teachers to be significant for their mental health and academic performance. One student stated that his high school did not provide them with the necessary support. No connections were maintained by any teacher. Another student considered having a continuous connection with the school counsellor to be the best thing the school could have done.

*The school’s role was minimal during this period. We were entirely isolated and our connections with teachers were completely cut when the educational process stopped for a month. Unfortunately, the school did not fulfil its expected role of supporting and educating us.* (S4)

Another student cited a negative impact on the motivation to study. The lack of proper follow-up and guidance might have reduced the students’ willingness to keep up with their studies.

*The remote-learning method did not attract me. I was undisciplined and used to quickly becoming bored. I did not study seriously or finish my homework because of the gaps and problems with this method. Therefore, I was neither motivated nor sufficiently aware of the importance of my study.* (S5)

#### School measures for COVID-19 prevention

**Fig 10:**
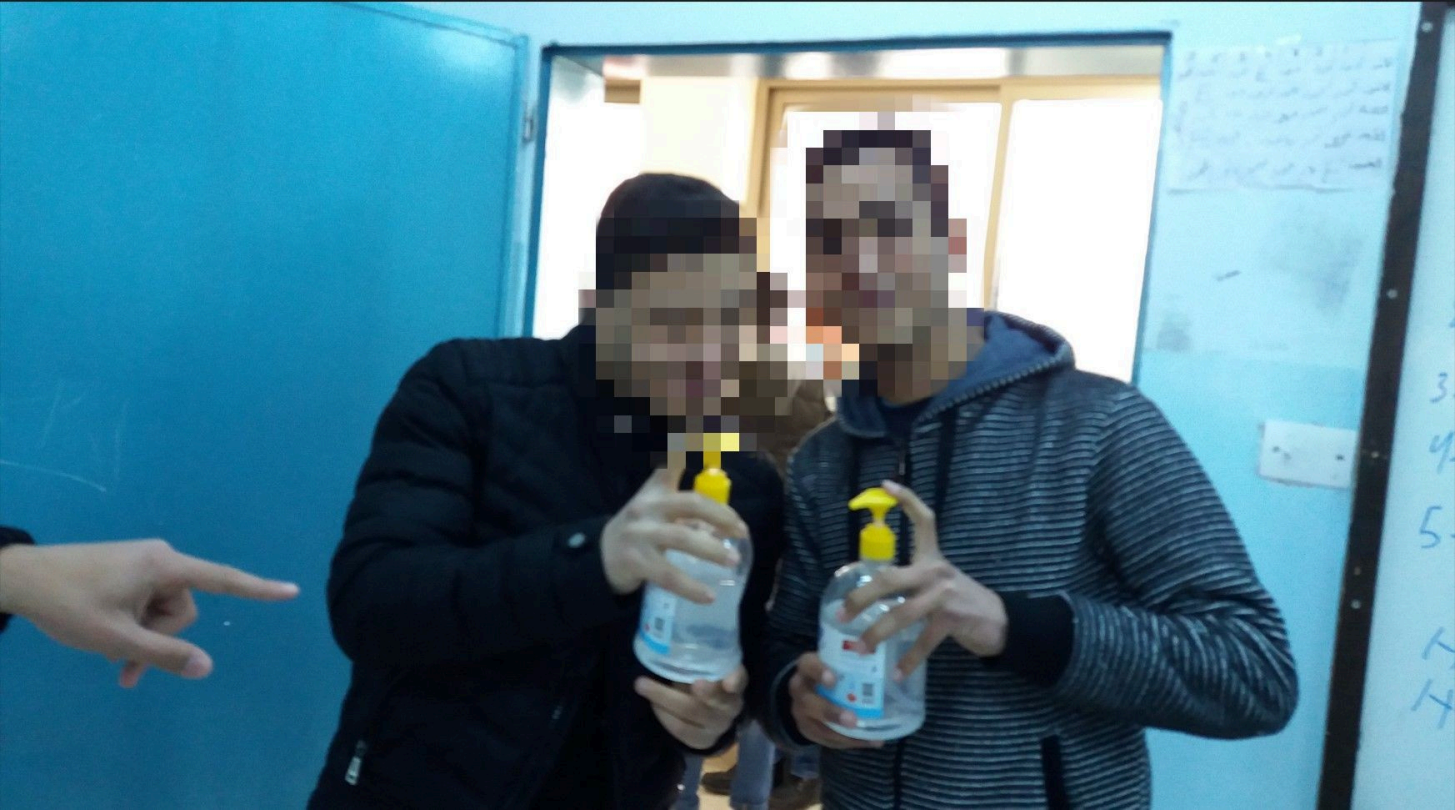
Students carrying hand sanitisers located in their classrooms. They were not wearing face masks.

**Fig 11:**
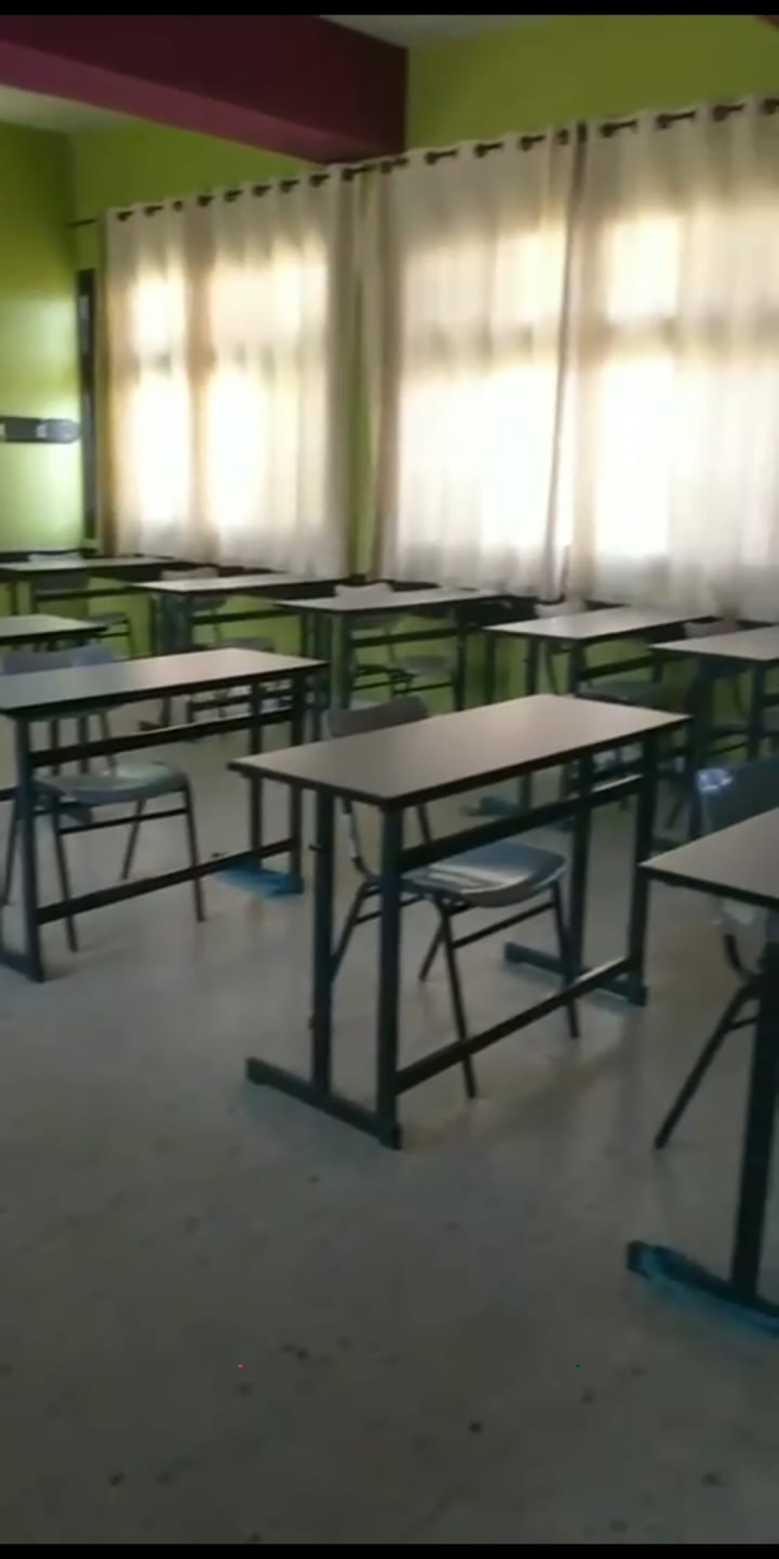
Empty classroom during the lockdown.

Some students reported that schools had implemented certain measures to help prevent the spread of COVID-19, such as providing sanitisers in classrooms and closing and implementing remote teaching (Figs 10 and 11). However, several students reported that they were not satisfied with the role played by schools. The reasons for this varied as several students reported that their schools did not do anything to raise their awareness of the pandemic or prevent its spread. A few students stated that their schools shared only social media posts to provide relevant information regarding the pandemic. One student reported that they were obliged to wear masks while their teachers did not, which made them feel careless about COVID-19.

*We did not take COVID-19 seriously and did not care about any preventive behaviour. Undoubtedly, the school was a partner in this carelessness. They did not enforce any actions. They obliged us to wear masks that were limited to students and not teachers. This significantly increased their conviction of not taking the pandemic seriously.* (S9)

Other students reported that their schools created a suitable and healthy environment for learning and preventing the spread of COVID-19. One student reported that his school administration had changed the classroom seating system (e.g. giving each student a single seat). Furthermore, the administration divided the large classes into two separate groups and imposed strict rules on wearing masks and maintaining a record of temperature. Another student stated that her school provided clear instructions to her and her siblings on what to do after her father was infected.

*Regarding the school’s role, they initially advised us to isolate ourselves and refrain from attending school after learning about our situation, even though not all courses were available online at that time. Nevertheless, they insisted that we stay at home and refrain from socialising. They also provided detailed instructions on the procedures.* (S10)

#### Transitioning towards remote learning

**Fig 12:**
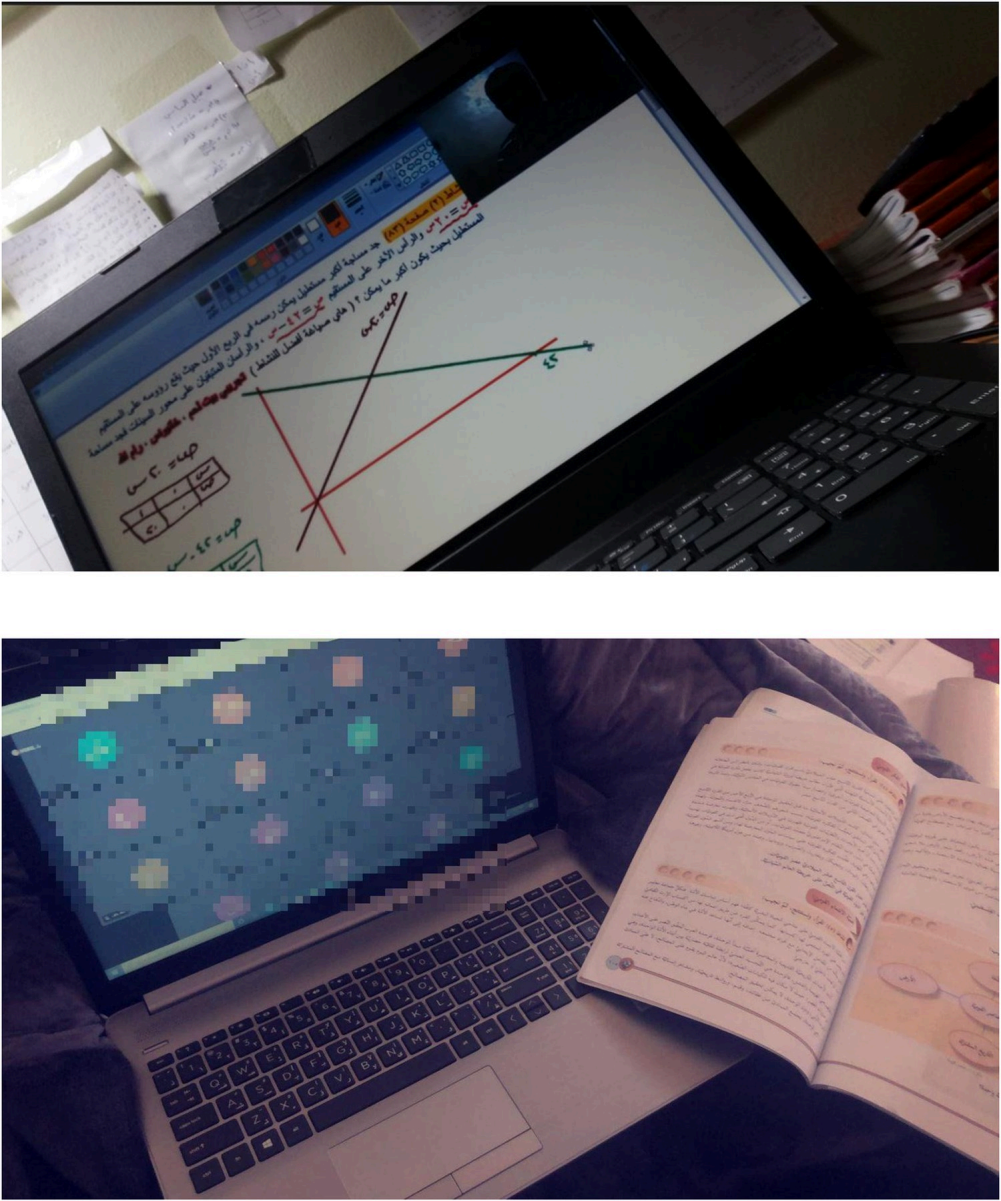
*Upper*: Student attending a mathematics class online. *Lower*: Student attending an online class, while reading from a textbook.

**Fig 13:**
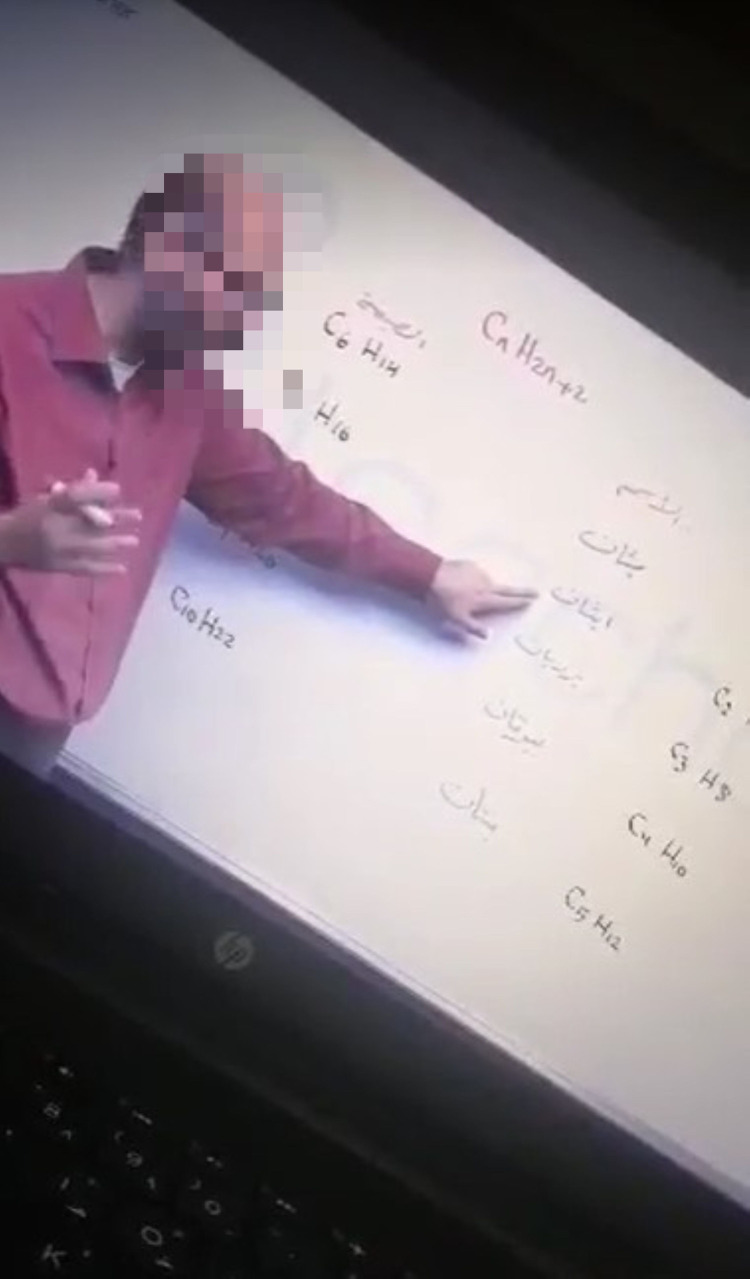
A recorded lesson to be watched by students at their convenience.

Schools had shifted from regular teaching methods to remote learning (Figs 12 and 13). Students had mixed opinions about remote learning during the pandemic. On the positive side, several students considered remote learning to be an opportunity for them to continue learning and studying and seek knowledge available online despite the lockdown. Remote learning introduced students with a way to stay connected with others and study and work together without the need to be physically available. Additionally, it provided them with a means of accessing various sources and opening their minds to self-study and development. Consequently, remote learning can positively impact their academic performance and increase their learning opportunities if conducted properly.

*Distance learning had a significant impact on my academic performance and achievements as it increased my eagerness to learn new things and refer to many books and sources.* (S8)

Several students reported that access to recorded lessons that could be reviewed was important for better academic performance (Fig 12). In addition, students agreed that remote learning saves time, which is usually spent in physically attending school.

*I think that distance learning can be highly effective if conducted properly as it saves time and effort during daily commutes to and from schools.* (S8)

However, students described several drawbacks of remote learning, such as technical challenges. For example, students complained about the lack of experience of teachers and themselves when using online platforms. They also complained of frequent disconnections due to either electricity outages or poor Wi-Fi networks. Another major problem was the insufficient availability of electronic devices in households.

*There are several problems that accompany this type of learning, including a lack of Internet access, teachers’ inability to deal with computers and students’ lack of electronic devices. Given these circumstances and obstacles, students experienced many academic weaknesses.* (S3)

Another challenge was students’ concerns about the limited communication between them and their teachers, which made education less effective in gaining information and understanding the teaching material. Furthermore, remote learning made it impossible for students to determine the trustworthiness and reliability of the sources used in their studies and the information they receive.

*Online learning was not very effective at the time and we did not benefit much from it. However, during the final exams, we were asked to attend school physically, which made it better for us to study with our classmates and teachers. Before that, online learning was not beneficial, and we did not properly understand the lessons.* (S10)

**Fig 14:**
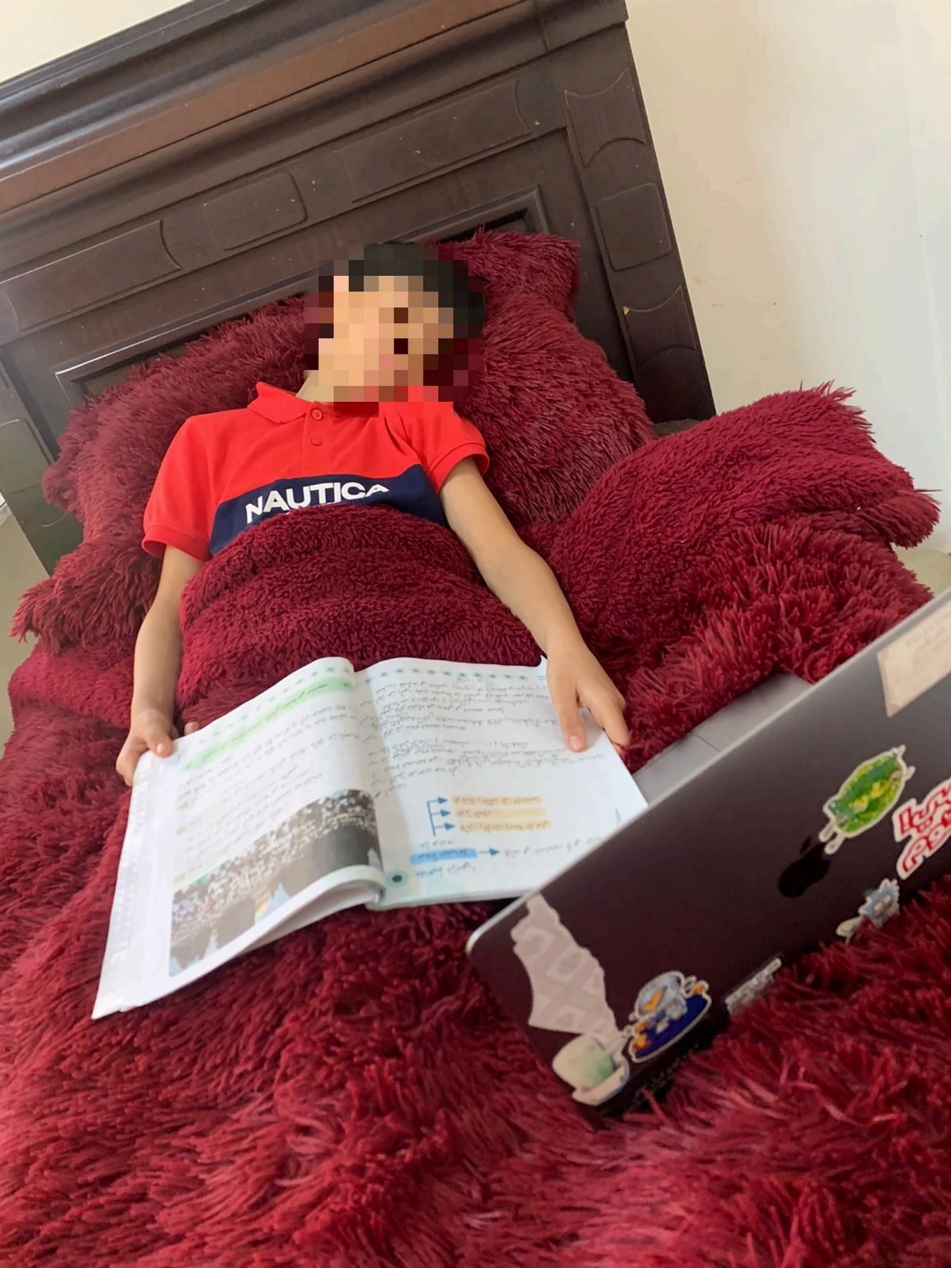
A student sleeping while studying.

Finally, students suffered from many side effects of this learning/teaching method at the individual level, such as laziness and a lack of motivation towards learning (Fig 14).

*I did not take remote learning seriously and was unable to engage in it. I quickly became bored and undisciplined. This lack of interest led me to neglect my studies and failure to complete my homework. Consequently, I lacked motivation and failed to appreciate the importance of studying and staying on track.* (S5)

Moreover, remote learning negatively influenced students, both mentally and socially. Students experienced stress when dealing with technical issues associated with remote learning. This may have contributed to the difficulties in keeping up with and understanding the taught material. Furthermore, they felt lonely, possibly due to their isolation from everything they knew, including their limited interaction with friends and teachers.

*In addition, remote learning significantly affects students’ psychological and social status during their long stay at home, causing loneliness and isolation from the world. It greatly influenced my academic, psychological and social skills.* (S12)

Students shared their thoughts and suggestions for avoiding the negative impacts of remote learning in the future. It was suggested that students should receive an orientation to learn ways to deal with the online learning process. This is also applicable to teachers who require orientation and training in using computers and specific software to conduct effective classes. Teachers and schools must adapt and modify their teaching methods to become more interactive and sustain students’ attention during the learning process. Another student suggested broadcasting educational sessions on local TV channels, covering the material for each grade.

*To learn from our experiences, remote learning must be enhanced. This may involve the use of innovative and interactive teaching methods to capture students’ attention. Measures, such as using cameras for better engagement, motivating students, revisiting online class content, and enhancing teachers’ online teaching skills, can contribute to improving students’ past experiences.* (S12)

Finally, one student emphasised the necessity for teachers and students to have strong communication skills to maintain continuous interaction and sustain an effective remote learning process.

*In my opinion, distance learning is effective if both the student and teacher are professionals and have excellent communication skills and if appropriate tools are available to convey information clearly.* (S9)

#### Relations among subthemes according to the theoretical framework

**Fig 15:**
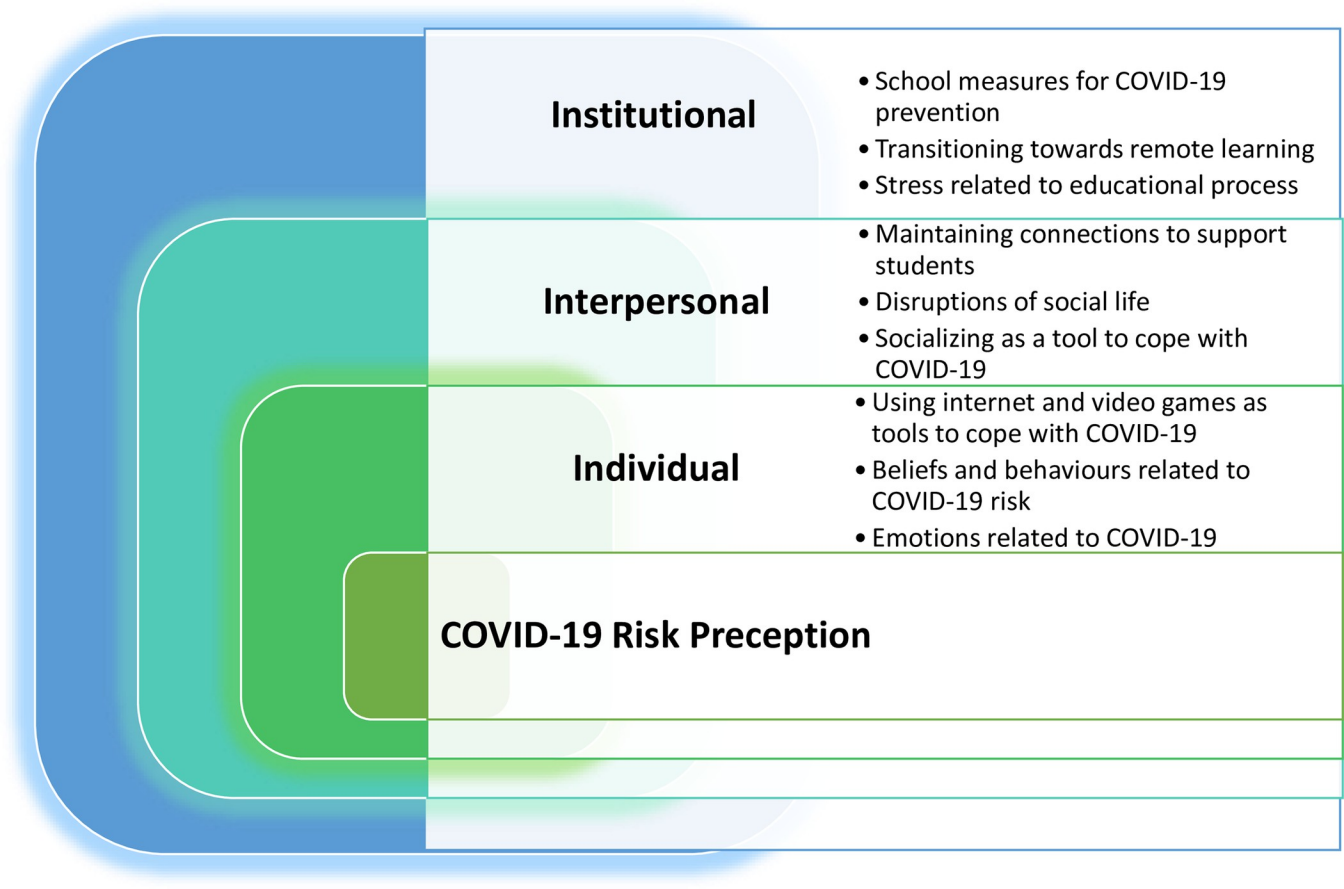
The classification of various factors according to the components of the theoretical framework.

The subthemes were organised based on the theoretical framework of this study, which was adapted from the social ecological model (Fig 15). Factors such as emotions, beliefs and behaviours related to COVID-19 are at the Individual Level. Playing video games as a coping mechanism can be a part of the individual level, while socializing as a coping mechanism can be included under the next level, which is the interpersonal level. This level includes the impact of disruptions to social life and the school’s role in maintaining connections to support students. However, the latter can also be subsumed under the institutional level, given that students expect such connections and support to be provided by schools. The institutional level addresses schools’ responses to the pandemic, represented by preventive measures imposed by schools and the transition towards remote learning. It also incorporates stress associated with the educational system and its demands.

## Discussion

The present study explored undergraduate students’ risk perceptions of COVID-19 and remote learning during their high school years. The content analysis revealed four themes as the main factors contributing to high school students’ risk perceptions of COVID-19. The first theme was psychosocial factors associated with the pandemic, including emotions, isolation and stress. The second theme focused on the mechanisms used to cope with the pandemic. The third theme discussed beliefs and behaviours that either increased or decreased the COVID-19 risk. The final theme addressed schools’ responses to COVID-19, including factors such as maintaining connections with schools, preventive measures and the transition towards remote learning.

This study emphasised emotional and mental well-being and specific coping mechanisms (e.g., socialising and gaming) as core aspects of students’ COVID-19 risk perceptions. The findings enrich the available models and frameworks that explain risk perceptions during the pandemic. Similarly, several studies reported or followed frameworks that explain factors contributing to the COVID-19 risk perception [47–49]. Some of these frameworks and models emphasise the role of cognitive factors (i.e., knowledge and awareness), emotions, mental health factors, and personal experiences (risk-increasing or mitigating behaviours) in the COVID-19 risk-perception [47, 48]. Li and Yu emphasised in 2022 on the role of schools in providing social support to students [49]. Our study adds to this by emphasising the critical role of schools in shaping students’ COVID-19 risk perceptions. According to our results, it can be inferred that the school’s role touches the core of risk perception by responding effectively to the pandemic and ensuring the implementation of preventive measures (i.e., remote learning), providing accurate information and proper guidance on responsible behaviours and providing emotional and social support systems. Therefore, schools’ actions can aggravate or mitigate students’ risk perceptions of the pandemic and other crises.

The COVID-19 pandemic has had a profound impact on students’ lives. For example, the pandemic has considerably affected their mental health. They experienced a range of negative emotions driven mainly by their fear of transmitting the disease to their loved ones. In a photovoice study in Bolivia, 12–16 years old adolescents reported sadness and fear of infecting their family members during the pandemic [39]. Adolescents and young adults in China were anxious about becoming infected [50]. Our results emphasise the significant impact of the pandemic on the well-being and mental health status of youth, indicating the need to address their concerns and provide adequate support.

Social isolation was the main reason for students’ suffering during the pandemic. This isolation might have led them to experience negative emotions such as fear, anxiety and stress. In Palestine, 57.7% of adolescents had fewer social connections after the pandemic and felt that their future was less promising than it was before the pandemic [36]. Such emotions may require mental health care referrals [51]. However, there is a lack of information and evidence regarding the mental and psychological health services provided to adolescents during the pandemic in Palestine [36].

The school’s role in supporting students elicited mixed reactions. Some students complained about limited support and communication with their teachers, whereas others complemented their school’s attempts to adapt to the pandemic. These results are particularly important when accompanied by additional negative emotions and stress originating from preparing for national high school examinations (Tawjihi) during the pandemic. Passing this examination is a determinant for their academic future and university admission [52]. Consequently, they spend most of their time throughout the year preparing for exams [53]. Considering their importance, all educational activities were suspended in Palestine, except for Tawjihi [54]. Even without the pandemic, Tawjihi poses tremendous pressure on students and has been linked to mental health problems such as anxiety and depression [53]. Given the possible synergistic impact of Tawjihi and COVID-19, our results indicate the necessity of maintaining connectedness with schools, and establishing strong teacher-student communication during crisis. This is supported by previous literature that has reported that such a relationship would assist in addressing students’ social and emotional needs and protecting their health and well-being from deterioration [55, 56].

The students in our study reported several strategies to cope with the stress caused by the pandemic. One of these activities involved spending time with friends and neighbours, which implied their eagerness for social connections and recklessness towards the COVID-19 risk. Nevertheless, maintaining social connectedness alleviates the impact of stress on health [57, 58]. Additionally, it is important to improve mental health and well-being by obtaining necessary social capital and support. In Palestine, almost half of the young adults consider higher social support to be correlated with lower stress, anxiety and depression [36]. Another coping mechanism was escaping by surfing the Internet. In our study, some students reported coping with the situation by using the Internet for self-development and learning new skills such as programming. In Russia and Turkey, students have reported positive attitudes towards online learning as a tool for personal growth [59].

Our study showed that engaging in entertainment and online gaming might provide an attractive escape from the difficult situations caused by the pandemic. However, rather than eliminating loneliness, it can quickly turn into an additional layer of isolation that impacts their social life. In a systematic review, several studies showed increased gaming time and addiction levels among children and adolescents during the pandemic [60]. Isolation and loneliness are the main drivers of Internet addiction [60, 61]. Increased gaming and social media hours among youth are coping mechanisms that mitigate psychological pain and prevent social isolation [60, 62]. However, social media use and media addiction has been associated with reduced well-being [49, 62]. This highlights the importance of social capital and support, as adolescents need guidance and support from their parents and families to survive these difficult conditions.

A subset of students in this study had a negligent attitude towards the seriousness of the pandemic and ignored the recommended preventive measures. Other studies have shown that adolescents and young adults were less compliant with social distancing than older adults [63–65]. This may explain some students’ noncompliance in our study.

Students’ relaxed attitudes towards preventive measures may also stem from their poor understanding and knowledge of the seriousness of the pandemic. During the H1N1 flu pandemic, perceived knowledge influenced adherence to recommended preventive practices in China [18]. Knowledge can indirectly influence individuals’ intentions to adhere to recommended measures through factors such as risk perceptions and attitudes [17]. On the other hand, another group of students in our study demonstrated responsible behaviour and recognised their role in preventing the spread of the disease to susceptible family members and the community. This emphasises a sense of civic responsibility which means that adolescents realise their active roles in their communities [66]. According to the participants in this study, schools played a vital role in shaping their experiences and attitudes during the pandemic.

Satisfaction with school efforts varied among students. Teachers’ inconsistencies and reluctance to wear masks while insisting students to do so affected their perceptions of their schools’ responses to the pandemic. In Turkey, students used metaphors such as risky areas, contamination centres and ‘house of horrors’ to describe the concept of school during the pandemic [67].

Students reported different reactions to remote learning, with some highlighting the advantages of accessibility and opportunities for self-learning and development. It was comforting for some students to have control over when they accessed the teaching materials, whether written or recorded in videos. Autonomy is essential for academic performance. Appropriately conducted remote learning may positively influence students’ academic performance. Similar results have been reported by Iglesias-Pradas et al. [68]. However, it is unrealistic to conclude that this approach is superior to traditional face-to-face approaches.

Some students had negative perceptions about remote learning. This finding is supported by previous studies on remote learning in Palestine. Palestinian schools face many challenges due to the low preparedness of all players in the educational process, including teachers, parents and students. Along with the lack of necessary skills, there is a lack of technical and logistical abilities, especially those related to high-quality devices, computers and internet connections [15]. The sudden change in educational models from face-to-face to online learning may have reduced the acceptance. Students need an adjustment period to develop a positive attitude towards online learning [13, 14].

Moreover, many obstacles associated with remote learning have been raised, such as technical hurdles, both at home and in school. In Indonesia, it was difficult for school teachers to prepare online lessons because of their limited capacity to provide online teaching [14]. In Canada, students reported facing technical issues with videos and Wi-Fi connections, which disrupted their learning experiences [13]. Our results indicate that not all families can provide devices for their children to attend online classes. Limited access to devices and Wi-Fi can be attributed to this digital divide. This became evident after switching to remote learning during the COVID-19 pandemic. This digital divide is associated with social inequity and inequality, which reduces accessibility to education for at least 50% of students worldwide [69].

Furthermore, remote learning perception was negatively influenced by poor communication with school staff, particularly teachers, and associated with social isolation. Widiasih et al. [14] reported similar results after interviewing mothers who raised concerns about poor bonding between students and their teachers and the loss of friendships. According to some students in our study, remote learning was unattractive, and they lost the motivation to attend classes. Fewer motivated students and teachers may experience remote learning failures [70]. Monotonous teaching methods used in online classes may have contributed to this finding [14]. Referring to our study, teachers must adopt interactive teaching methods to effectively engage students in the learning process. Without strong social interaction and engagement between teachers and students, the remote-learning process is less effective than traditional teaching methods [70].

Finally, it is essential to discuss the COVID-19 pandemic by considering the context of chronic exposure to violence among Palestinians. Contrary to our expectations, the students who participated in this study did not mention violence as one of the factors related to their COVID-19 experience. The combined burden of violence exposure and the pandemic presented a unique challenge. However, experiencing the pandemic was an unprecedented event that students had never experienced. Therefore, their attention may have shifted towards the most immediate threat. In a Libyan study, college students reported that the civil war complicated the COVID-19 situation [22]. Regardless of the type of violent event, this applies to the Palestinian situation. Another reason for the current results is that we did not directly investigate violence or its association with COVID-19. Further studies are required to address this issue.

### Strengths and weaknesses

This study has several strengths. The use of the photovoice approach and WhatsApp made it easier and more convenient for university students to express their perceptions in a visually accessible format. Additionally, it highlighted the long-term impact of distance learning and COVID-19 on students going through important periods in their lives. Another strength was the diversity of the sample, including students of both sexes, various faculties and study disciplines and their hometowns, which enabled us to have a better understanding of the challenges faced by students in an important transition phase in their lives during the COVID-19 crisis.

On the other hand, this study has several limitations. The dropout rate was high, which may have an impact on the depth and variability of the data. However, this was mitigated by using a diverse sample. According to the students, their reasons were busy schedules, hesitation to share contact information, especially female students, and inadequacy of the reward for participating in this study. Students lacked the willingness to perform additional tasks along with their studies, which they considered an overburden. Moreover, having a stranger ask students to participate in a research project and share their contact information to perform certain tasks, such as answering questions and taking photos, could have created confusion and doubts. Latz reported similar concerns [71], who described possible reasons for students’ lower motivation to participate in a photovoice study. Latz [71] also mentioned that only 15 of the sixty-five approached students agreed to participate, and only seven attended the interviews. We tried to overcome students’ hesitancy to participate by offering voluntary hours later as an incentive, which might have helped us obtain photos from the students. Various recruitment techniques were employed to overcome this challenge. Furthermore, students had the freedom to use written or voice messages to deliver their responses.

Another limitation related to the use of WhatsApp or emails is missing real-time interactions with the students and deeper probing and exploration of students’ views by researchers. Additionally, focusing mainly on students’ experiences during the early stages of the pandemic may have been affected by recall bias. However, giving students the time to consider their responses before submission might have a positive impact on the quality of their responses by overcoming the lack of real-time interactions and recall bias.

## Conclusion

The present study highlighted the massive mental health impact experienced by students during the COVID-19 pandemic, including fear, stress, anxiety and feelings of isolation. Furthermore, it highlighted the different coping strategies that students used to alleviate their distress. They also expressed varying levels of risk perception regarding COVID-19 and remote learning. Additionally, it highlighted the central role of schools in the COVID-19 risk perception among students, Emphasising the importance of designing and implementing health promotion interventions and programs that address the specific needs of students during future health crises, such as the COVID-19 pandemic, within educational institutions.

Therefore, a comprehensive strategy is required to address mental health and educational issues. The key measures for improving students’ overall well-being and educational experiences during and after the pandemic include improving communication between students and educational institutions, providing a well-functioning and strong support system, and providing equal access to distance learning materials by investing in remote learning infrastructure and teacher training to enhance student engagement.

In the future, it is critical to learn from these experiences and apply them to policies and practices to increase the quality of service provided to students during crisis, which often occurs in Palestine. As revealed in the present study, Palestinian students’ adaptability offers hope if their needs are effectively identified and addressed.

## Supporting information

S1 Checklist COREQ (COnsolidated criteria for REporting Qualitative research) checklist. (PDF)

S1 Table Characteristics of study participants. (PDF)
