# Supplementary material for: Palestinian university students’ perspectives on COVID-19 risk and remote learning during the pandemic: A qualitative photovoice study
Source: PLoS One. 2024 Oct 11;19(10):e0311972. doi: 10.1371/journal.pone.0311972 (PMC11469486; doi:10.1371/journal.pone.0311972)
Supplement: S1 Table — (PDF) [file pone.0311972.s002.pdf]

Supplementary Table 1: Characteristics of study participants.

Student	Age	gender	Specialty	Year at university	Residence
**S1**	20	Female	Nutrition and Dietetics	Second year	Ramallah
**S2**	19	Female	Nutrition and Dietetics	First year	Ramallah
**S3**	18	Male	Computer science	First year	Hebron
**S4**	20	Male	Cyber security	First year	Ramallah
**S5**	19	Female	Nutrition and Dietetics	First year	Ramallah
**S6**	20	Male	Electrical engineering	Second year	Ramallah
**S7**	20	Male	Physics	Second year	Ramallah
**S8**	20	Male	Computer engineering	Second year	Ramallah
**S9**	20	Male	Computer science	Second year	Ramallah
**S10**	19	Female	Translation	Second year	Ramallah
**S11**	20	Male	Computer science	Second year	Ramallah
**S12**	19	Female	Nutrition and Dietetics	First year	Ramallah
**S13**	19	Female	Audiology and Speech Therapy	First year	Jerusalem
**S14**	20	Male	Computer science	Second year	Ramallah
**S15**	20	Male	Accounting	Second year	Ramallah
**S16**	19	Female	Marketing	Second year	Ramallah
